# High-dose-rate plesiotherapy with customized molds in non-melanoma skin cancer: efficacy and safety at 10 years—single institution experience

**DOI:** 10.1007/s12094-021-02718-2

**Published:** 2021-10-26

**Authors:** I. Membrive Conejo, O. Pera Cegarra, P. Foro Arnalot, A. Reig Castillejo, N. Rodríguez de Dios, X. Sanz Latiesas, G. Deza, J. Quera Jordana, E. Fernandez-Velilla Cepria, A. Martínez Moñino, F. Liu Cheng, M. Algara López

**Affiliations:** 1 Radiation Oncology Department, Hospital del Mar, Parc de Salut Mar, Passeig Marítim 25, 08003 Barcelona, Spain; 2 Institut Hospital del Mar d’Investigacions Mèdiques Barcelona, Barcelona, Spain; 3 Pompeu Fabra University, Barcelona, Spain; 4 Dermatology Department, Hospital del Mar, Parc de Salut Mar, Barcelona, Spain; 5 Universitat Autónoma de Barcelona, Barcelona, Spain

**Keywords:** Skin cancer, Plesiotherapy, Brachytherapy, Custom molds

## Abstract

**Purpose:**

Our center adopted high-dose-rate brachytherapy with surface applicators (plesiotherapy) in 2008, creating custom molds to treat irregular areas. This study describes the efficacy and safety outcomes after extensive follow-up in the patients.

**Methods/patients:**

We planned the treatment using two computed tomography (CT) scans: the first to delineate the lesion and the second after placing the thermoplastic mold. Fusing the two CT images enables planning of the target volume and pinpointing, where the catheters are in the mold.

**Results:**

Seventy patients received plesiotherapy, either exclusively or following excision in patients with risk factors for recurrence. Those receiving plesiotherapy alone showed a complete response rate of 95.8%, and recurrences occurred in 5.7% at a mean follow-up of 96.2 months. Chronic toxicity appeared in 26.6% of patients, but severity was limited to grade 1 or 2.

**Conclusions:**

High-dose-rate brachytherapy with customized molds yields a high rate of complete response, with long-term recurrence rates in line with similar studies and an acceptable toxicity rate.

## Introduction

Non-melanoma skin cancer is the most frequently diagnosed cancers in our population. In 2018, there were 1,042,056 diagnosed cases and 65,155 deaths worldwide [1]. The incidence of this type of tumor is increasing in both older people, due to lengthening life expectancy, and in younger people, as a result of greater exposure to the sun and other behavioral changes [2, 3].

Treatment options for pre-malignant or superficial lesions follow different strategies, including topical treatments, cryotherapy, and surgery [4]. For invasive lesions, therapy mainly consists of surgery or radiotherapy, chosen depending on factors, such as comorbidities or the desired aesthetic outcome [5]. Indeed, aesthetic sequelae may be important, as the scars resulting from excision, especially in prominent areas, change the person’s physical appearance and may negatively impact the patient’s interactions with their environment [6, 7]. At times, both treatment modalities are used, for instance if tumor-free margins are not achieved in surgery or risk factors for recurrence are detected in the surgical specimen [8]. One factor that plays into the decision on treatment modality is the location of the lesion: for tumors on the face, radiotherapy, used alone or as an adjuvant therapy, is quite common, as the aesthetic impact is lower than with surgery alone and good outcomes are obtained [9]. Recently, the role of systemic treatments (especially the use of hedgehog pathway inhibitors) is also being assessed in the context of neoadjuvant or definitive treatments in certain types of lesions that cannot be treated surgically or with radiotherapy initially due to their size, infiltration or number. [10].

The radiotherapy techniques used vary according to the size, depth, and location of the lesion. Large lesions are usually treated with electron beams from a linear accelerator, whereas smaller lesions are treated with photons generated by kilovoltage units or with plesiotherapy [11].

Plesiotherapy is based on establishing contact between the high-dose-rate (HDR) radioactive source and the lesion. There are diverse kinds of applicators, depending on the tumor site and the availability at each treatment center, for example round applicators with differing diameters (Leipzig, Valencia) or silicone applicators with built-in catheters (Freiburg Flap) [12].

For lesions in irregular areas (mostly in the nasal or ear area), it is difficult to ensure proper coverage using flat applicators, because in some targeted areas, the applicator cannot be placed correctly, or some regions would be underdosed. In these cases, customized molds are made; different centers have developed different techniques, all yielding good outcomes for both efficacy and safety.

The treatment is administered on visible cancer lesions with an adequate safety margin to try to eradicate adjacent microscopic disease. Defining the target volume using computed tomography (CT) is also recommended for the 3D dose calculation to delimit the at-risk organs (mainly the eye) and optimize treatment [13]. Skin lesions are generally very superficial and difficult to delimit on CT images, so radiopaque markers are placed on the skin as an aid. However, this solution causes a dosimetric problem, as the radiopaque markers interfere with the dosimetry.

Our department launched its HDR brachytherapy unit in 2008, and one of the main purposes was to perform plesiotherapy in patients with skin cancer. Since the adoption of this technique, the unit has set the objective of treating lesions in irregular areas using customized molds.

The aims of the present study are to describe the workflow and methodology for the creation of customized molds and planning via CT, and to describe the efficacy and toxicity outcomes at 10 years from the implementation of the treatment technique.

## Materials and methods

### Patient selection

Patients are evaluated in a multidisciplinary board made up of specialists in oncology, radiotherapy, dermatology, plastic surgery, maxillofacial surgery, and otolaryngology. During the board meeting, members agree on the best treatment option for the individual patient, and the patients who are candidates for radiotherapy are referred to our service, where the medical radiation physics team decides on the most appropriate radiotherapy technique to use.

During the first visit, the target volume is delimited visually, by palpation and, if necessary, with the help of a dermatoscope, which helps to define the edges of the tumour more precisely in cases, where the edges are poorly delimited visually to ensure adequate margins. If necessary, this can be done with the help of the dermatologist. A photograph of the lesion is taken to favour reproducibility.

This study includes the data for all patients treated from September 2008 (the first use of the technique with customized molds) to February 2018.

### Creation of the mold

**Fig. 1:**
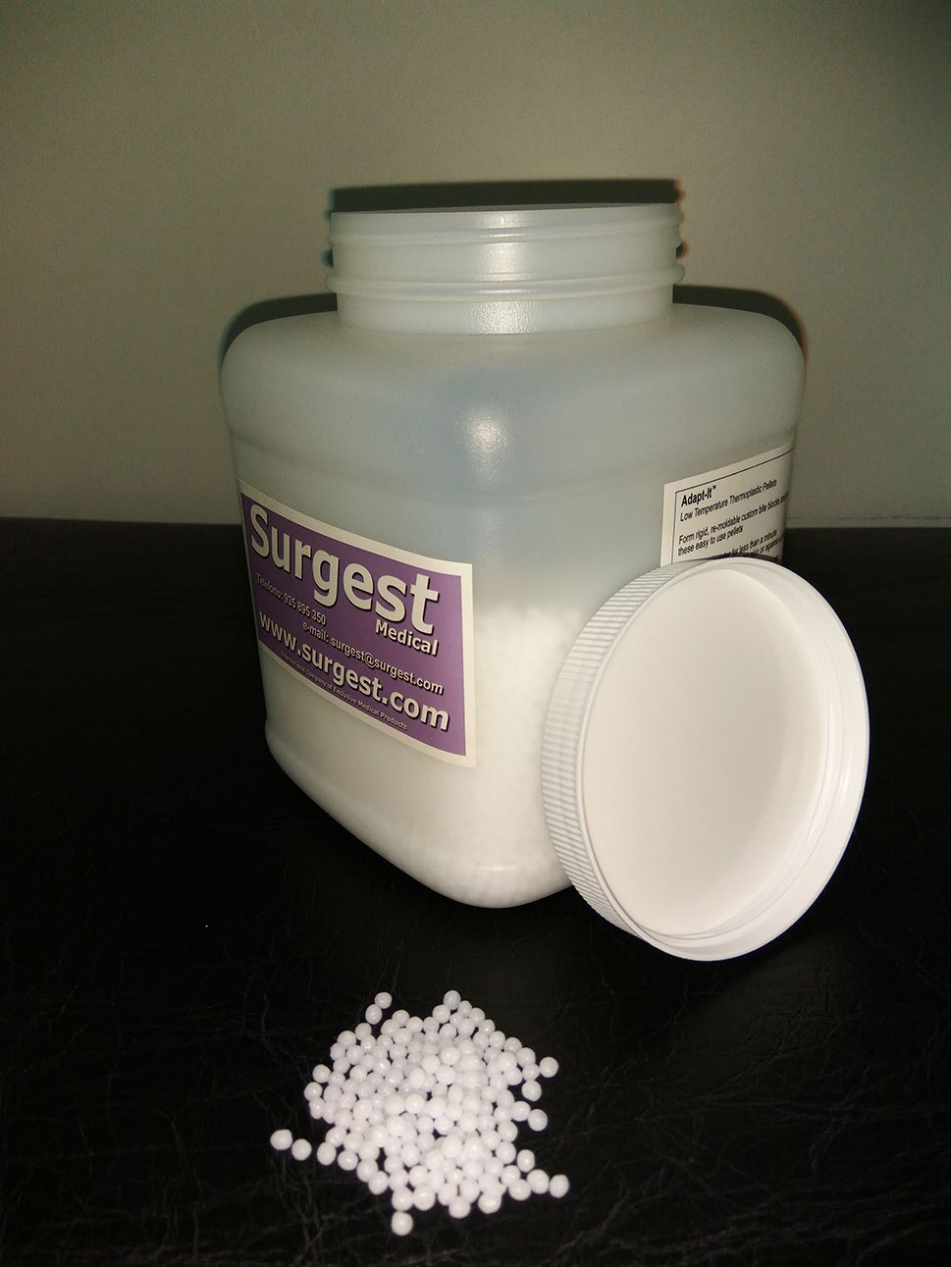
Thermoplastic material for the creation of customized molds

**Fig. 2:**
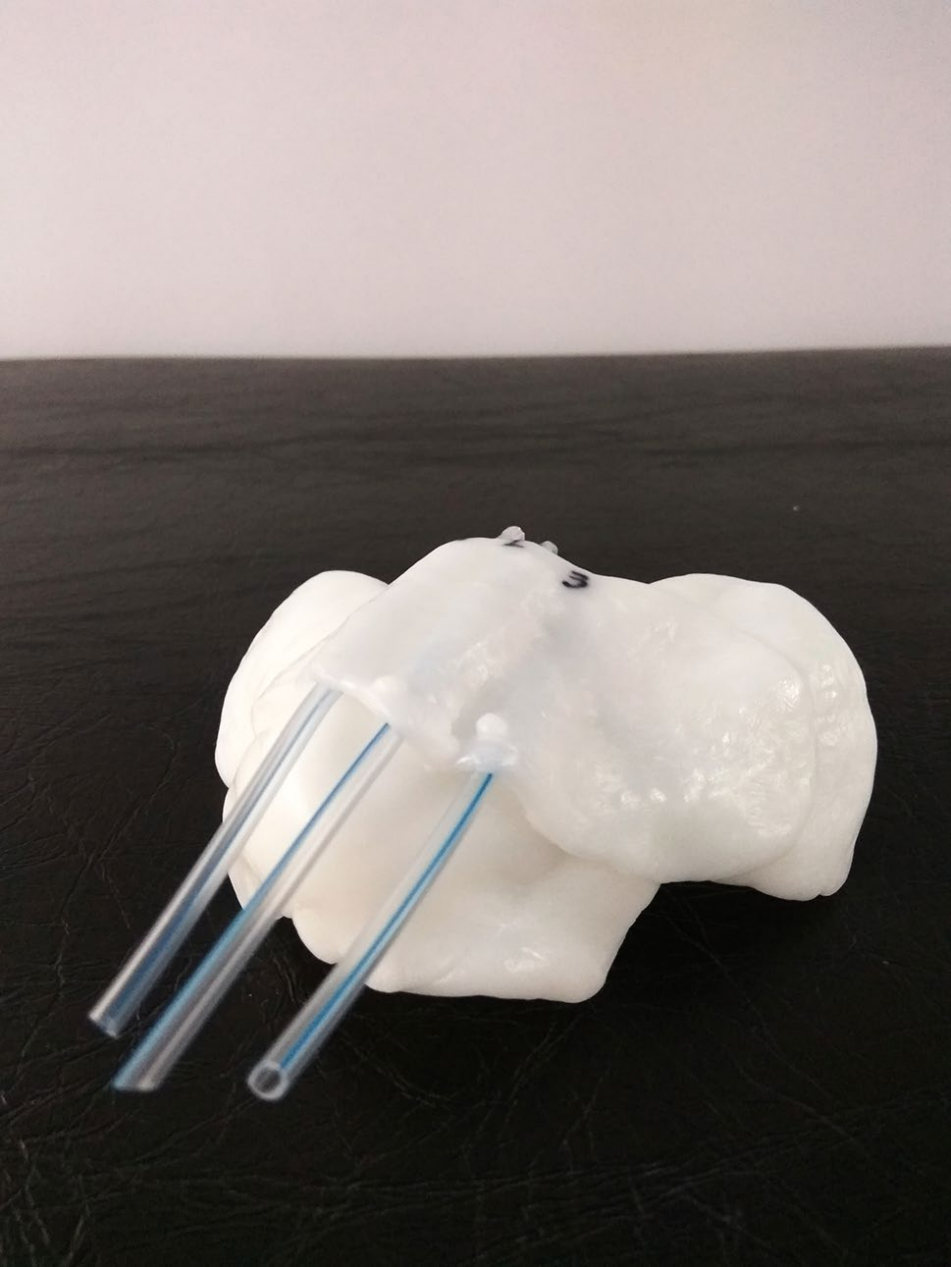
Customized mold, ready for initiation of treatment

**Fig. 3:**
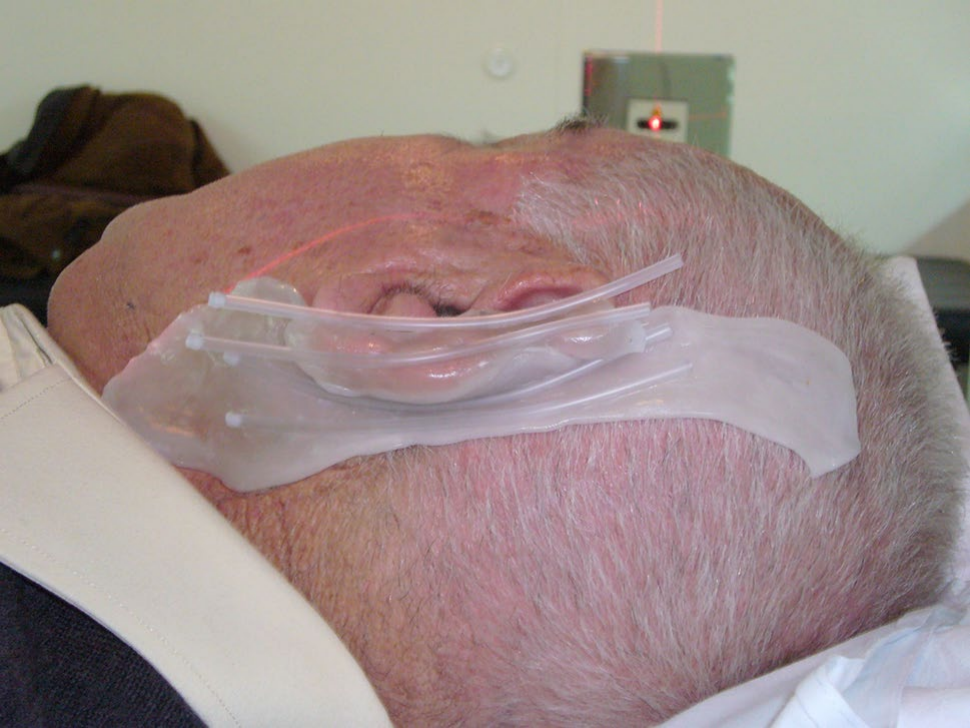
Customized mold, placed in the patient

The custom molds are made from small balls of thermoplastic material (Adapt-it Surgest Medical; Fig. 1), which are heated to 70 °C to make them malleable enough to adapt to any anatomical surface. First, a 5 mm layer of this material is created, ensuring that no air is present between the material and the treatment surface. Then, plastic tubes with a diameter of 3.3 mm (we use the nasogastric probe, size CH10) are placed on top. For the treatment, catheters containing the radioactive source will be inserted into these tubes, which must be placed in parallel 1 cm apart, covering the entire lesion to be treated (Figs. 2, 3).

We have encountered different limitations related to the creation of the mold:

- The patient must remain immobile for a considerable amount of time, while the mold is being made.
- At times, the mold does not perfectly adapt to the patient surface, with air present in some areas that complicates the dosimetry calculation.
- The placement of the catheters may not be equidistant (with some proximate areas that produce hot zones during the dosimetry), or in some cases the distance to the surface may be irregular.
- Following placement of the catheters and performance of the dosimetry, the treatment may not be optimal, making it necessary to re-make the molds.

### Treatment planning

Once the mold has cooled down and is totally solid, the simulation with CT can proceed. The CT unit used is the Somaton Confidence CT (Siemens Healthcare) system, producing images with 1 mm slices. The patient undergoes the first CT with their head immobilized by a thermoplastic mask. During the scan, radiopaque markers are used to delimit the lesion with an adequate safety margin, usually 3–5 mm, depending on the size and location of the lesion. A second CT is then performed without the markers but with the customized mold.

**Fig. 4:**
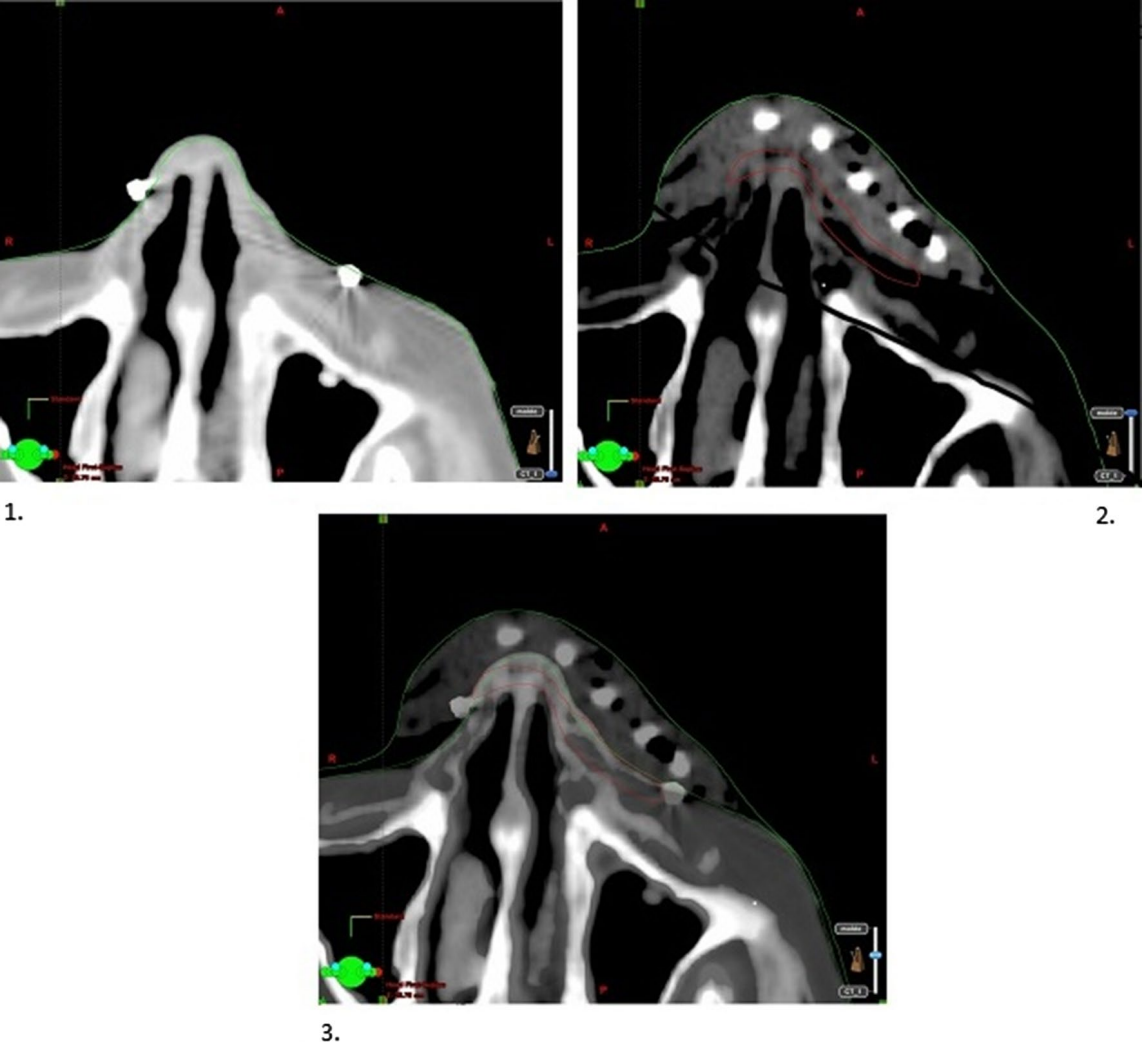
1—Computed tomography (CT) image with radiopaque marker in place. 2—CT image with customized mold in place. 3—Fusion of images for treatment planning

The delimitation of the target volume is carried out using Brachyvision software (v8.1 Varian Medical Systems). The two series of CT images are overlaid to visualize the radiopaque markers from the first onto the second CT. These images will be used to delimit the target volume, with the aid of the radiopaque markers, and subsequently the at-risk organs (Fig. 4). Thereafter, the medical radiation physicist performs the dosimetry calculation on the series of images without the radiopaque markers, determining the positions that the radioactive source should be in as well as the stop times to ensure the correct coverage of the target volume. An isodose of 90% should cover at least 95% of the volume, and the surface dose should be no more than 150%. The dose that at-risk organs receive should likewise not exceed the predefined thresholds of each.

### Treatment administration

Treatment is administered with the Gammamed plus iX HDR brachytherapy unit (Varian Medical Systems) with an Ir192 source. The treatment regimen proposed depends on the patient’s general condition and any difficulties they may have in getting to the radiation oncology unit. For patients in stable conditions and without major barriers for accessing the service, a total dose of 54 Gy is administered, in fractions of 3 Gy every other day. In frail patients, whose mobility may be more limited, a total dose of 40 Gy is administered in fractions of 4 Gy per session, also on alternate days. Throughout the treatment, secondary toxicity is monitored on a weekly basis.

### Outcomes assessment

Acute and chronic toxicity are evaluated according to the common terminology criteria for adverse events (CTCAE v.5.0) [14]. These assessments were undertaken jointly with the dermatology service, weekly over the course of the treatment, biannually for the first 3 years, and annually thereafter. The follow-ups also served to check for recurrence on the treated lesion; in case of suspected persistence or relapse, biopsy or excision was used to confirm the diagnosis. The data analysis was performed retrospectively to determine the complete treatment response rate and disease-free survival.

## Results

### Patient and treatment characteristics

From September 2008 to February 2018, 70 patients received HDR surface brachytherapy with customized molds. Table 1 presents a summary of the characteristics of the patients and their lesions. Mean age was 79 years (range 63–91). All the lesions treated were classified as stage 1 according to the 8th TNM-UICC staging system and had a depth of 5 mm or less. Patients for whom there were clinical doubts regarding the depth of the lesion underwent an ultrasound during a joint visit with a dermatologist. In 60 cases (85.7%), the histology was basal cell carcinoma; in 9 (12.8%), squamous cell carcinoma; and in 1 (1.5%), lentigo maligna. Twenty-five (35.7%) patients had undergone prior surgery on the lesion, and adjuvant brachytherapy was performed due to the presence of risk factors (positive margins and/or perineural invasion). Most lesions were located in the nasal area (80%), followed at some distance by the pinna (11.4%), periorbital area (5.7%), and cheek (2.9%).

**Table 1:** Patient characteristics (N = 70)

Variables	Frequency
Gender	
Men	48.6% (34)
Women	51.4% (36)
Age in years, mean (range)	79 (63–91)
Lesion site	
Nose	80% (56)
Periorbital area	5.7% (4)
Ear	11.4% (8)
Cheek	2.9% (2)
Histology	
Basal cell carcinoma	85.7% (60)
Squamous cell carcinoma	12.8% (9)
Lentigo maligna	1.5% (1)
Prior surgery	
Yes	35.7% (25)
No	64.3% (45)

Treatments were performed according to three different regimens, with a lower dose per fraction in the first patients treated following adoption of the technique and larger fractions in more recent years. Early on, patients followed a scheme of 33 fractions of 2 Gy on consecutive days; subsequently, patients received 18 fractions of 3 Gy or 10 fractions of 4 Gy, depending on the patient’s mobility and their ease in traveling to our center. Sessions were scheduled either daily or on alternate days, according to patient preferences.

### Toxicity during treatment

Skin toxicity was assessed during the course of treatment according to the skin ulceration scale of the CTCAE v.5.0; we observed grade 1 toxicity in 14.2% of the patients, grade 2 toxicity in 64.2%, and grade 3 toxicity in 21.6%. No patient presented grade 4 toxicity, nor was it necessary to suspend any treatments because of toxicity. When topical treatments were necessary, the nursing staff provided them in our service. Following our department’s protocol, skin integrity impairments were treated with occlusive dressings, changed every 24–48 h. We continued to dress wounds in our service following the finalization of the treatment, and patients were followed until toxicity was resolved.

### Chronic toxicity

To assess chronic toxicity, we used the skin atrophy, hyperpigmentation, hypopigmentation, and induration scales of the CTCAE v5.0. Grade 1 skin atrophy was observed in 12.3% of the cases; grade 1 hyperpigmentation, in 2%; grade 1 hypopigmentation, in 16.8%; grade 1 induration, in 5%; and grade 2 induration, in 3.8%. No chronic toxicity was reported in 73.4% of the patients.

### Efficacy

Patients were followed for a mean 96.2 months (range 7–156). At 3 months, after completion of the brachytherapy, our service and the dermatology service jointly assessed treatment response, observing a complete response rate of 95.8% in the patients treated exclusively with plesiotherapy (i.e., who had not undergone surgery). Three patients presented a partial response and completed their treatment with a simple excision, with no need for reconstructive surgery. Two of these lesions were in the alae nasi, and one on the nasal dorsum; the histological type was squamous cell carcinoma in two and basal cell carcinoma in one case.

Four recurrences (5.7%) were observed in the treatment field over follow-up, none of which occurred in the patients who needed surgical rescue after plesiotherapy. Two patients with recurrent lesions of only a few millimeters in size (both in the alae nasi) were treated with a simple excision and did not need reconstructive therapy. The other two, with lesions on the ala nasi and nasal dorsum, underwent excision and flap reconstruction. In all four cases of recurrence, the histology was squamous cell carcinoma. At the time of writing, all of these patients were free of disease.

No cases of recurrence were recorded among the 25 patients who were treated with adjuvant brachytherapy due to the presence of risk factors. Likewise, there were no cases of regional or distant recurrences in our series.

## Discussion

Currently, the gold standard treatment for localized skin cancer is surgery, in the form of simple excision with direct closure or in some cases using flap reconstruction or skin grafts. In some cases, surgical treatment may not be appropriate, generally due to patient comorbidities, as the highest incidence of these lesions is in older patients, who often present severe frailty, making surgery ill-advised [2]; or due to the location of the lesion, because surgical interventions on facial sites (where incidence is highest) can cause aesthetic alterations that limit patients’ quality of life [5]. In other cases, adjuvant radiotherapy is recommended due to the presence of certain risk factors, such as positive margins or perineural invasion [15]. The NCCN guidelines reflect this role of radiotherapy in both squamous and basal cell carcinoma. Radiotherapy (either external radiotherapy or brachytherapy depending on the characteristics of the lesion) is considered in inoperable patients, or in adjuvant treatments with the presence of risk factors, although these treatments should always be avoided in patients under 60 years of age if possible. Systemic treatment should also be considered in locally advanced cases in which local treatment is not possible or with a large number of lesions, and there are promising results with the use of hedgehog pathway inhibitors [16, 17].

In the multidisciplinary skin cancer unit of our center, these patients are offered the possibility of receiving radiotherapy. For lesions of less than 4 cm in diameter and an invasion depth of less than 5 mm, contact brachytherapy, also known as plesiotherapy, is indicated. Brachytherapy techniques have been used to treat skin cancer for more than a century [18], but in recent years, it has become increasingly common, as a growing body of evidence supports its efficacy and safety [19]. These studies report excellent local control, which varies from 83.3 to 98% or even 100% of cases, depending on the scheme used and the tumor histology, site, size, and depth [20].

Arenas et al. [21] observed a rate of disease-free survival of 94.5% at 5 years after using Leipzig applicators, and of 88% at 5 years using customized molds, with grade 4 acute toxicity of less than 2.2%.

Other publications also report the utility and efficacy of customized molds (Table 2). Vavassari et al. [22] assessed patients treated for lesions on the eyelid with customized molds made of thermoplastic material for a mean follow-up of 51 months. The authors did not observe any local recurrences and only one case of chronic toxicity, of grade 2 according to the radiation therapy oncology group (corneal ulcer).

**Table 2:** Characteristics of studies of customized brachytherapy molds

Study	*N* patients	Type of applicators	Treatment scheme	Response rate (%)	Recurrence	Toxicity
Arenas et al. (2015) [21]	134	Wax and plastic	45–54 Gy for basal cell carcinoma 45–57 Gy for squamous cell carcinoma 3 Gy per fraction	98	Disease-free survival of 88% at 5 years	Grade 4 < 2.2%
Vavassari et al. (2019) [22]	10	Thermoplastic	30–48 Gy 2–4.5 Gy per fraction		0% at mean follow-up of 51 months	Grade 2, *n* = 1
Maroñas et al. (2011) [23]	51	Wax	48–57 Gy 3–4 Gy per fraction		9.8% at mean follow-up of 45 meses	Grade 1–2, 78% Grade 4, 21%
Kalaghchi et al. (2018) [24]	60	Alginate	30–52 Gy 3–4 Gy per fraction	95.2	2.8%, brachytherapy alone 11.1%, as adjuvant therapy	Grade 1–2, 43% at 1 year from end of treatment
	70	Thermoplastic	40–66 Gy 2–4 Gy per fraction	95.8	5.7%, brachytherapy alone 0%, as adjuvant therapy	Chronic grade 1, 22.8% Chronic grade 2, 3.8% No toxicity, 73.4%

Maroñas et al. [23] described their experience treating patients with custom molds made of wax. Their group used this technique on 51 tumors, administering a dose of 48–57 Gy in fractions of 3 Gy or 4 Gy, applied three times a week. At a mean follow-up of 45 months, five recurrences were observed, with the patients undergoing rescue surgery. Kalaghchi et al. [24] reported a complete treatment response rate of 95.2% at 3 months after treatment with custom molds made of alginate; just 2.8% of the patients treated exclusively with brachytherapy had a recurrence, compared to 11.1% of those treated adjuvantly following surgery. There were no cases of long-term toxicity of grade 2 or higher. Studies including a greater number of lesions confirm the efficacy and safety of this type of treatment; for instance, Olek et al. [25] analyzed 273 lesions treated with customized applicators (made of thermoplastic material), observing a recurrence rate of 4.8% at 25 months and a low rate of chronic toxicity (erythema 4.4%, chronic ulceration 4%).

The present study reports our experience in the long term, describing outcomes in an elevated number of patients treated with customized molds. This technique was developed to optimize treatment for small tumors located in irregular anatomical sites. We decided to do the planning using CT to precisely define the positions of the catheters embedded in the mold, ensure the correct dose distribution, and limit the dose in at-risk organs. Our analysis of local recurrence and toxicity shows the efficacy and safety of this technique. The toxicity we observed can be attributed to the high surface dose reached. It generally did not provoke symptoms in the patient because of its superficial nature, and it was self-limiting, with full resolution shortly after completing treatment.

The treatment schemes used with these techniques vary in different centers, because, since the efficacy is high, fractionation is adapted to the organization of each service. Currently, the tendency is to administer shorter treatments (hypofractionation) to make them more comfortable for patients of advanced age.

Other hypofractionation schemes have been described as well. For example, Jumeau et al. [26] treated 11 patients with wax or silicone applicators, administering: 25 Gy in 5 fractions postoperatively; 30 Gy in 6 fractions in patients receiving brachytherapy alone; and 8 Gy in a single session in palliative care. The local control at 2 years was 91%, while radiodermatitis of grade 1 was observed in 50% of the patients, and of grade 2 in 33%.

In our center we used different fractionation schemes, following the recommendations of the Groupe Européen de Curiethérapie (GEC) and the European SocieTy for Radiotherapy and Oncology (ESTRO) [27] according to the patient’s age and general condition. Typically, the regimen was 54 Gy in fractions of 3 Gy, administered three times weekly. A longer fractionation scheme was also used, 66 Gy in daily fractions of 2 Gy, with the aim of improving the long-term cosmetic outcome in young patients. In contrast, very old patients in poor general health were given 40 Gy in 10 fractions to favor their comfort.

## Conclusions

High-dose-rate plesiotherapy is an efficacious and safe treatment. Unlike other techniques (electrons, surface applicators), it can be used on small skin cancers located on irregular surfaces. The use of these techniques is a clear example of a personalised oncological treatment. The treatment yields a low rate of local recurrence, and toxicity is superficial and does not have a clinical impact in the patient.

## Data Availability

The data sets generated during and/or analysed during the current study are available from the corresponding author on reasonable request.
